# Transparent Cellulose Nanofibrils Composites with Two-layer Delignified Rotary-cutting Poplar Veneers (0°-layer and 90°-layer) for Light Acquisition of Solar Cell

**DOI:** 10.1038/s41598-020-58941-4

**Published:** 2020-02-06

**Authors:** Weihua Zou, Zhangheng Wang, Delin Sun, Xiaoqin Ji, Pingfang Zhang, Zhihong Zhu

**Affiliations:** Central South University of Forestry and Technology, Shaoshan South Road 498, Changsha, 410004 China

**Keywords:** Energy science and technology, Materials science

## Abstract

Our transparent cellulose nanofibrils composites (TCNC) directly from rotary-cutting poplar veneer (RPV) whose lignin can be easily stripped by our treatment. This TCNC is prepared by stripping lignin of original RPV and infiltrating epoxy resin (ER) into delignified RPV. This TCNC with two-layer delignified RPVs whose grains perpendicular (0/90°) to each other, which were solidified on solar cell while infiltrating ER. This TCNC with high transmittance (~90%), high haze (~90%), and equal refractive index fluctuation. Comparing with epoxy resin (ER), this TCNC can enhance open circuit voltage (VOC) from 1.16 to ~1.36 and short circuit density (JSC) from 30 to ~34 for the solar cell, and can enhance test fore from 0.155 kN to ~0.185 kN and displacement from 43.6 mm to ~52.5 mm.

## Introduction

Wood is a kind of abundant organic macromolecule resource on the earth. Wood has excellent material properties which including high strength, high toughness, high modulus, low density, low thermal conductivity, biodegradability, sustainability and so on^1–3^. The contents of wood mainly include cellulose and hemicellulose (≈70%), lignin (≈30%), and its cellulose and hemicellulose are colourless substance^4,5^. New wood functionalization approaches have made it possible to combine load-bearing and functional properties in biobased wood structures^6–12^. After stripping lignin or chromogenic groups, a kind of transparent wood composites (TWC) can be prepared by infiltrating the cavity of wood with a polymer^3,4,13–16^. The TWC with high transmittance and high haze for light acquisition of solar cell, which have a significant enhancement in solar energy conversion efficiency^17–22^. Some researchers have prepared TWC for light management in solar cells^17,18^, and they focused on TWC from radial-cutting veneer for two reasons. First, radial-cutting veneer is easier than rotary-cutting veneer in delignification. Second, radial-cutting veneer has equal refractive index fluctuation, but rotary-cutting veneer hasn’t. However, rotary-cutting veneer could obtain far larger breadth from wood trunk to compare with radial-cutting veneer.

Farmed poplar is a kind of widely distributed agro-forestry tree species in many nations due to its fast growth rate, short rotation period, multiple uses and high economical value^23–27^. Reasonable use of farmed poplar can meet the human demand for TWC, and can avoid the consumption of natural forest resources^27^. Poplar has the characteristics of ultra-short fibers, and its rotary-cutting poplar veneer (RPV) is better than radial-cutting poplar veneer (RPV) in mechanical properties^28,29^.

**Figure 1:**
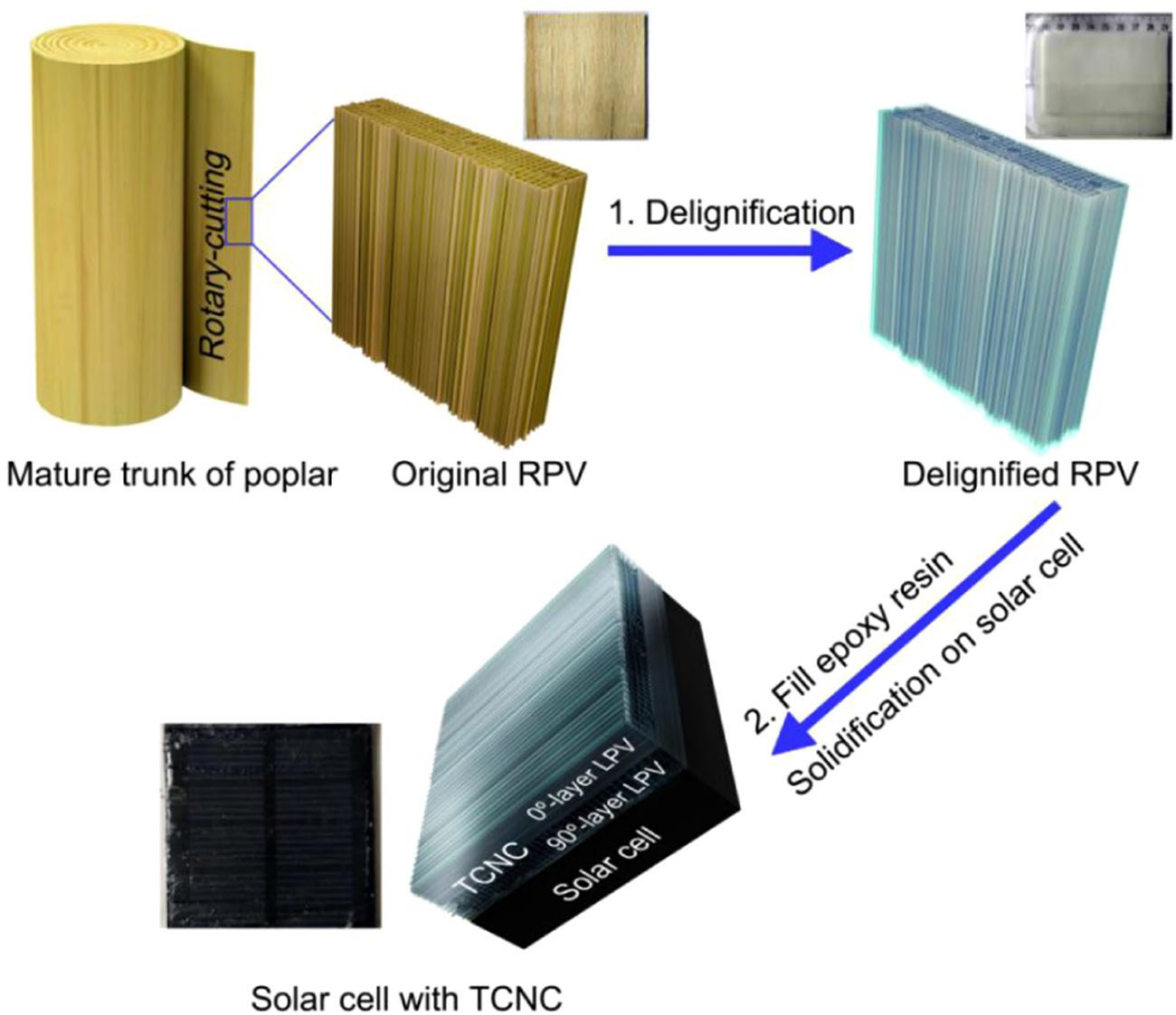
Original RPV was stripped lignin by our hydrothermal treatment and impregnation treatment. TCNC with two-layer delignified RPVs that were solidified on solar cell while infiltrating ER.

In our works, the kind of transparent cellulose nanofibrils composites (TCNC) directly from rotary-cutting poplar veneer (RPV) whose lignin can be easily stripped by our treatment. The RPV is from mature trunk of farmed poplar. This TCNC is prepared by stripping lignin of original RPV and infiltrating epoxy resin (ER) into delignified RPV. This TCNC with two-layer delignified RPVs whose grains perpendicular (0/90°) to each other, which were solidified on solar cell while infiltrating ER (Fig. 1). This TCNC is with high transmittance, high haze and equal refractive index fluctuation, which can enhance the light acquisition of solar cell.

In our previous work, the lignin of RPV was stripped by boiling in potassium hydroxide (KOH) solution (2.7 mol L^−1^ in deionized water) and immersing in sodium hypochlorite (NaClO) solution (0.81 mol L^−1^ in deionized water), polyurethane (PU) and its hardener (polyisocyanate) were infiltrated into the delignificated RPV^27^. In our this work (Table 1), the lignin of RPV was stripped by hydrothermal treatment in sodium hypochlorite (NaClO) solution, and impregnation treatment 1 in ammonium persulfate ((NH_4_)_2_S_2_O_8_) solution, and impregnation treatment 2 in sodium hypochlorite (NaClO) solution. Epoxy resin (ER) and its hardener were infiltrated into the two-layer delignified RPVs in this work. To compare with our previous work, this TCNC with more high transmittance (~90%), high haze (~90%) and equal refractive index fluctuation.

**Table 1:** The chemical formula and method for preparation TCNC.

Method	Chemicals (g, ml)	Temperature(°C)	Time(h)
Hydrothermal treatment	NaClO (30 g), deionized water (1000 ml)	130–160	3
Impregnation treatment 1	(NH_4_)_2_S_2_O_8_ (50 g), deionized water (200 ml)	15–25	72
Impregnation treatment 2	NaClO (30 g), deionized water (500 ml)	15–25	24
ER infiltration	ER (45 ml), its hardener (15 ml)	25–30	24

## Results and Discussion

### Cell wall contents of RPV before and after delignification

**Figure 2:**
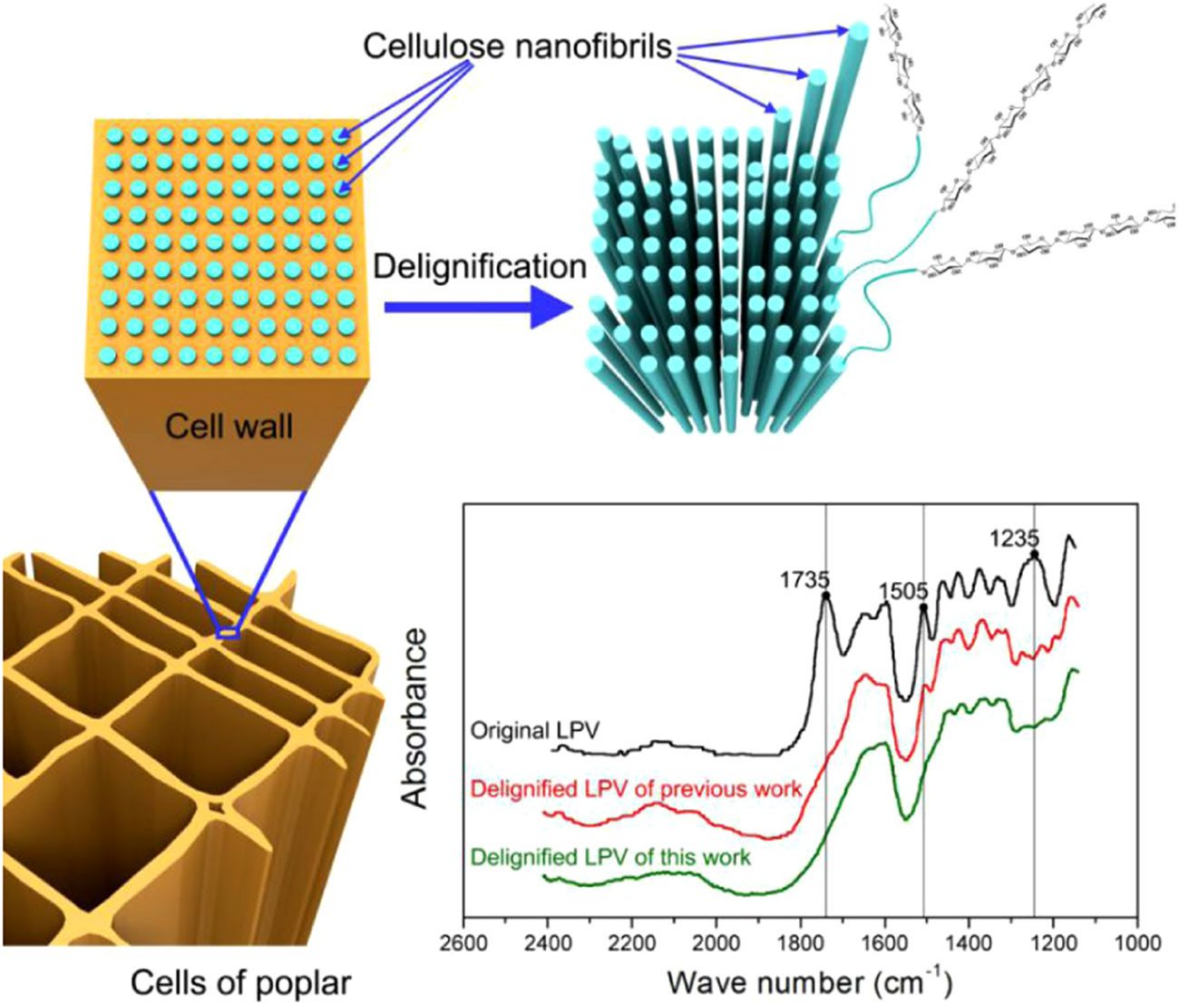
Graphical illustration and FTIR spectra for original RPV and delignified RPV.

Fourier transform infrared spectroscopy (FTIR) was used to investigate the changes of its cell wall contents from original RPV to delignified RPV by using FTIR-850 (Gangdong, Tianjin, China). In the FTIR spectrum, the band at 1505 cm^−1^ is aromatic compounds (phenolic hydroxy groups) and is attributed to aromatic skeleton vibrations from lignin^15,27,30^. The bands at 1235 cm^−1^ and 1735 cm^−1^ are characteristic of hemicelluloses and C=O functional group respectively^27,31–33^. Comparing with original RPV and delignified RPV of previous work (ref. ^27^), the peaks at 1505 cm^−1^, 1235 cm^−1^ and 1735 cm^−1^ have disappeared in delignified RPV of this work, proving that lignin, hemicellulose and C=O functional group have been stripped from original RPV in our this work (Fig. 2). As Table 2 shows, the absolute-drying weight of original RPV (60 mm × 60 mm × 3 mm) is about 2.124~2.381 g, and the absolute-drying weight of delignified RPV (60 mm × 60 mm × 3 mm) is about 1.041~1.164 g. After delignification, the absolute-drying weight of delignified RPV was about 50% of original RPV.

**Table 2:** The absolute-drying weight from original RPV to delignified RPV.

	Sample 1	Sample 2	Sample 3
The absolute-drying weight of original RPV (60 mm × 60 mm × 3 mm)	2.124 g	2.196 g	2.381 g
The absolute-drying weight of delignified RPV (60 mm × 60 mm × 3 mm)	1.041 g	1.074 g	1.164 g

### Microstructure of TCNC

**Figure 3:**
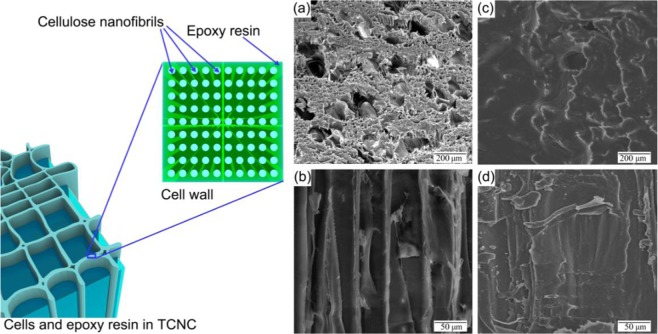
Graphical illustration and SEM images about TCNC. (**a**,**b**) are SEM images of radial direction and longitudinal direction from delignified RPV, respectively. (**c**,**d**) are SEM images of radial direction and longitudinal direction from TCNC, respectively.

ER is a kind of index-matching polymer for delignified wood, and transmittance of delignified wood can be developed by infiltrating ER^13^. Before and after ER infiltration, delignified RPV and TCNC were cut from its radial direction and longitudinal direction, these sections were examined by using Quanta 450 scanning electron microscopy (FEI, US). Figure 3(a–d) are SEM images of radial direction and longitudinal direction from delignified RPV and TCNC, respectively. In Fig. 3, graphical illustration and SEM images indicate that the microstructure of TCNC is well-infiltrated and well-preserved by ER.

### Optical properties of TCNC for light acquisition of solar cell

In TCNC, its cellulose nanofibrils network and its lumen are the main pathway of optical transmittance. Modification of the wood cell wall will help to tune the light scattering properties of its material, and introducing strong scattering, resulting in diffused luminescence from embedded quantum dots^15,16,27^. The optical haze of TCNC is due to its nature-structural anisotropy and its light scattering properties.

**Figure 4:**
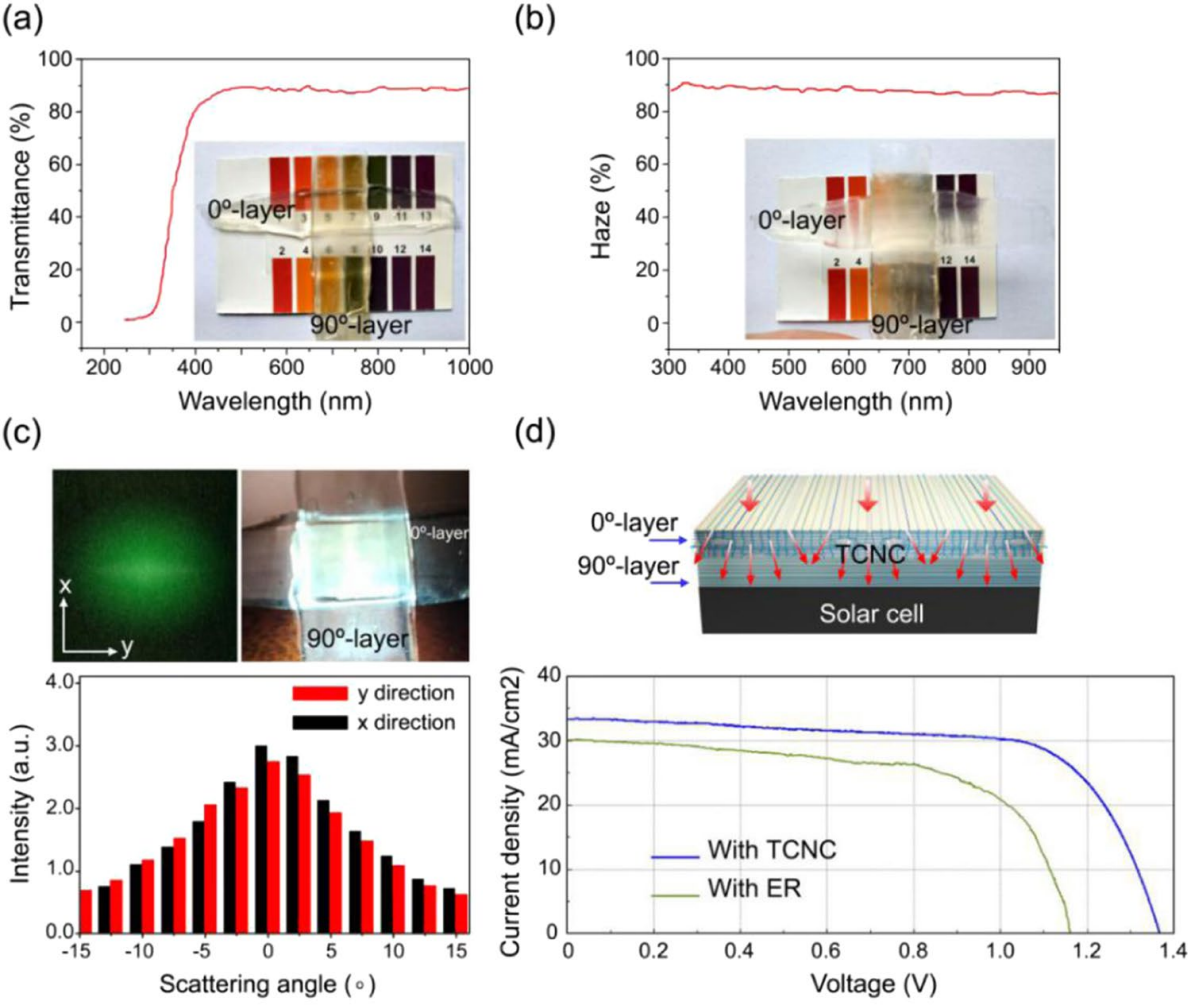
(**a**,**b**) This kind of TCNC with high transmittance of ~90%, high haze of ~90%. (**c**) In refractive index fluctuation, x direction close y direction. (**d**) The current density-voltage curves of solar cell with ER and with TCNC.

Transmittance and haze were obtained by using WGT-S transmittance and haze tester (SGIC, Shanghai, China). Figure 4(a,b) shows that our TCNC with high transmittance of ~90%, high haze of ~90%. When TCNC be in contact with the substrate whose colored shape can be clearly seen, and when it be took 5 mm above the substrate whose colored shape becomes very fuzzy.

S130C photodiode power sensor (Thorlabs, US) was used to record the scattered light intensity distribution in both the x and y directions on the surface of TCNC. Figure 4(c) indicates that this TCNC with almost equal refractive index fluctuation in the x and y direction. In our previous work, TWC with one-layer delignified RPV, that has anisotropic light diffraction and lower refractive index fluctuation in the direction of aligned cellulose fibers^27^. Our this TCNC with two-layer delignified RPVs whose grains perpendicular (0/90°) to each other, that making its refractive index fluctuation of the x direction close to the y direction.

According to its high transmittance, high haze and equal refractive index fluctuation, TCNC is superior transparent layers for light acquisition of solar cell, which as Fig. 4(d) shows. The electrical properties of solar cell mainly includes open circuit voltage (VOC) and short circuit density (JSC)^8^, and the current density-voltage curves of solar cell with ER and with TCNC were obtained by using CS310H electrochemical workstation (CorrTest, Wuhan, China). Figure 4(d) and Table 3 indicate that TCNC improving the light acquisition of solar cell to compare with ER, and enhancing the solar cell’s VOC from 1.16 to ~1.36 and its JSC from 30 to ~34.

**Table 3:** Open circuit voltage (VOC) and short circuit density (JSC) from solar cells with ER or TCNC, respectively.

	With ER	With TCNC 1	With TCNC 2	With TCNC 3
Open circuit voltage (VOC)	1.16	1.35	1.36	1.36
Short circuit density (JSC)	30	33.5	34	34.2

### Mechanical characteristics of TCNC

**Figure 5:**
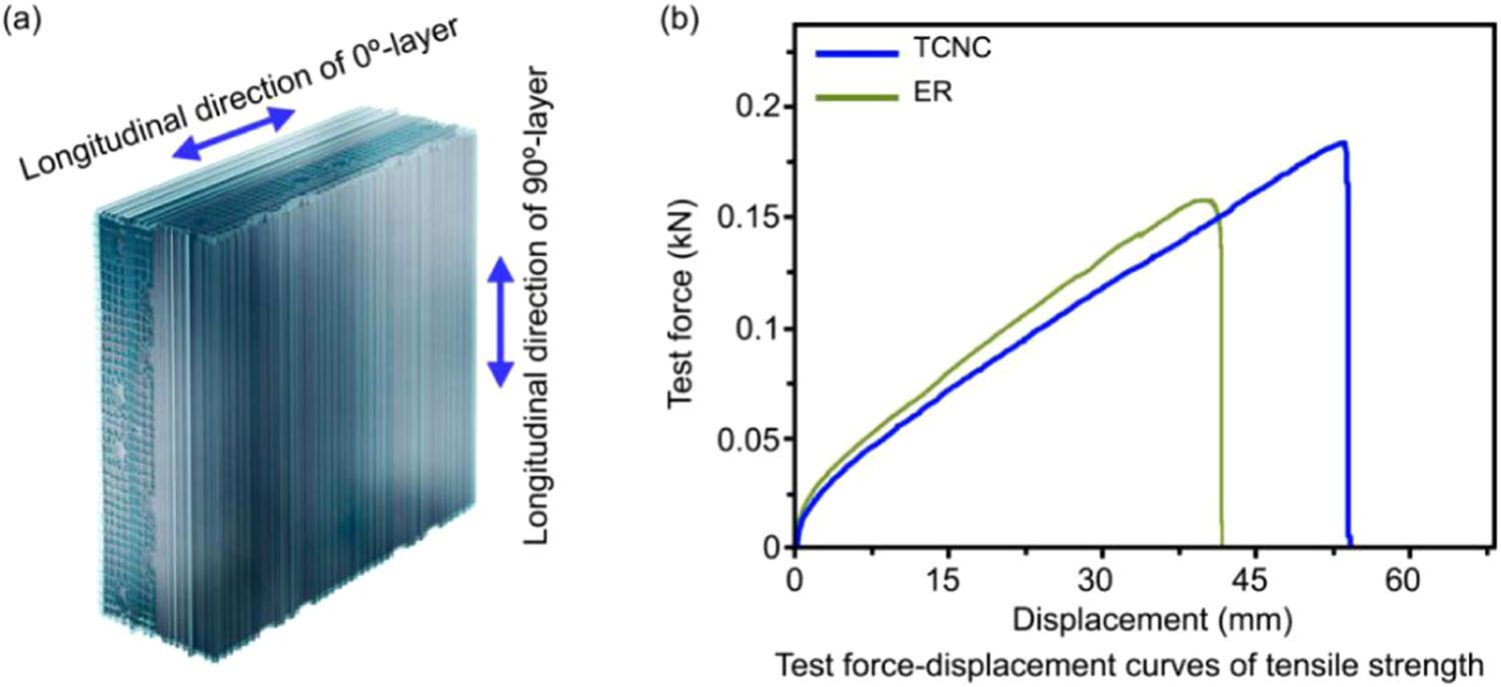
(**a**) Graphical illustration about 0°-layer close 90°-layer in tensile strength of longitudinal direction. (**b**) Test force-displacement curves of tensile strength about TCNC and ER.

ER that is a kind of current material for surface of solar cell at present, but our TCNC has better tensile strength than ER. Figure 5(a) indicates that TCNC has almost equal tensile strength from longitudinal directions in 0°-layer and 90°-layer. Comparing with ER (60 mm × 60 mm × 3 mm), the test fore of TCNC (60 mm × 60 mm × 3 mm) can enhance from 0.155 kN to ~0.185 kN, and its displacement can enhance from 43.6 mm to ~52.5 mm, which as Fig. 5(b) and Table 4 show. The tensile strength was tested by using the tester of mechanical property SmartTest (Joyrun, China). TCNC can meet more flexible shape for solar cell to compare with ER.

**Table 4:** Test fore and displacement from ER or TCNC, respectively.

	ER	TCNC 1	TCNC 2	TCNC 3
Test fore (kN)	0.155 kN	0.183 kN	0.185 kN	0.186 kN
Displacement (mm)	43.6 mm	52.2 mm	52.5 mm	52.9 mm

## Conclusions

For improving practicability of TWC in light acquisition of solar cell, we have basically mastered a kind of method of preparing TWC from original rotary-cutting poplar veneer. Our TCNC with high transmittance (~90%), high haze (~90%), and almost close refractive index fluctuation, which can enhance VOC from 1.16 to ~1.36 and JSC from 30 to ~34 for the solar cell to compare with ER. Although ER being a kind of current material for surface of solar cell at present, however, comparing with ER, our TCNC can enhance test fore from 0.155 kN to ~0.185 kN and displacement from 43.6 mm to ~52.5 mm, which can meet more flexible shape for solar cell. Furthermore, our future work will pay more attention to reduce the time cost and the resource consumption in preparation of TCNC, and to improve the quality of TCNC for the light acquisition of solar cell.

## Materials and Methods

### Materials and chemicals

Original RPV (60 mm × 60 mm × 3 mm) was purchased from Mudan Wood Co., Ltd. (Suqian, China). solar cell (Solar single cell of silicon, 54 mm × 54 mm) was purchased from Aike Electronic Technology Co., Ltd. (Ningbo, China). NaClO (>98%), (NH_4_)_2_S_2_O_8_ (>98%), deionized water, and ethyl alcohol absolute (C_2_H_6_O, >99.5%) were purchased from Aladdin Biochemical Technology (Shanghai, China). ER and its hardener were purchased from Wuhui Port Adhesive Co., Ltd. (Hangzhou, China).

This TCNC is prepared by stripping lignin of original RPV and infiltrating epoxy resin (ER) into delignified RPV, and the steps of delignification include hydrothermal treatment and impregnation treatment (1, 2), as Table 1 shows.

### Stripping lignin of original RPV

The step 1 of delignification is hydrothermal treatment that boiling the sample of original RPV in the NaClO solution (0.405 mol L^−1^ in deionized water) for about 3 h at 130–160 °C. Then, the RPV sample was took out from the solution and its chemicals was removed by rinsing in hot distilled water. The step 2 of delignification is impregnation treatment 1 that immersing the RPV sample in the (NH_4_)_2_S_2_O_8_ solution (1.1 mol L^−1^ in deionized water) for about 72 h at 15–25 °C. Then, the chemicals of sample was also removed by rinsing in hot distilled water. The step 3 of delignification is impregnation treatment 2 that immersing the RPV sample in the NaClO solution (0.81 mol L^−1^ in deionized water) for about 24 h at 15–25 °C until its color has disappeared. After stripping lignin, the delignified RPV was preserved in C_2_H_6_O.

### Infiltrating ER into delignified RPV and solidifying it on solar cell

First, the delignified RPV was attached to the surface of the sample of solar cell by C_2_H_6_O. Second, a kind of liquid resin was prepared by mixing ER and its hardener at a ratio of 3 to 1 (ER 45 ml, its hardener 15 ml), and this liquid resin (60 ml) was covered on the delignified RPV. Then, this liquid resin was filled into the delignified RPV by vacuumizing in RV-620-2 vacuum reactor (YBIF, Shanghai, China) at 25–30 °C. All the above processes should be completed within 30 min. After first layer of delignified RPV (0°-layer RPV) solidifying on solar cell for about 24 h at 25–30 °C, second layer of delignified RPV (90°-layer RPV) was solidified on 0°-layer RPV by repeating the above processes.
